# Factors Associated with Intubation After Less Invasive Surfactant Administration (LISA); A Single-Center Cohort from Saudi Arabia

**DOI:** 10.3390/children12091196

**Published:** 2025-09-08

**Authors:** Kamal Ali, Abdulghani Lodhi, Saleh S. Alqarni, Mohanned Alrahili, Mohamed Almahdi, Reem Alharbi, Rahaf Alshahrani, Monirah Alroshoud, Ahad Aldhafiri, Amal Alharbi, Maisa Alqahtani, Abdulaziz Homedi, Ibrahim Ali, Saif Alsaif

**Affiliations:** 1 Neonatal Intensive Care Department, King Abdulaziz Medical City-Riyadh, Ministry of National Guard Health Affairs, P.O. Box 22490, Riyadh 11426, Saudi Arabia; lodhiab@mngha.med.sa (A.L.); qarnis@ksau-hs.edu.sa (S.S.A.); alrahilimo@mngha.med.sa (M.A.); almahdimo2@mngha.med.sa (M.A.); alharbire6@mngha.med.sa (R.A.); alshaharanira@mngha.med.sa (R.A.); alroshoudmo@mngha.med.sa (M.A.); aldhafeeriah3@mngha.med.sa (A.A.); alharbiam11@mngha.med.sa (A.A.); alqahtanima11@mngha.med.sa (M.A.); homediab@mngha.med.sa (A.H.); ahmedia@mngha.med.sa (I.A.); saifs@mngha.med.sa (S.A.); 2 King Abdullah International Medical Research Center, Riyadh 11481, Saudi Arabia; 3 College of Medicine, King Saud Bin Abdulaziz University for Health Sciences, P.O. Box 3660, Riyadh 11481, Saudi Arabia

**Keywords:** LISA, surfactant, respiratory distress syndrome, prematurity, intubation, intraventricular hemorrhage, neonatal outcomes

## Abstract

**Highlights:**

**What are the main findings?**

- In a single-center cohort of preterm infants (26–34 weeks) receiving LISA as first-line surfactant (*n* = 41), 61% avoided intubation within 72 h, and higher gestational age predicted LISA success (aOR 1.44 per week; 95% CI 1.07–1.95).
- LISA failure was associated with greater odds of intraventricular hemorrhage (any grade; aOR 10.08, 95% CI 1.29–78.50) and longer NICU stay, while other short-term morbidities were similar.

**What is the implication of the main finding?**

- Bedside risk stratification is feasible: lower-GA infants may warrant closer monitoring and earlier escalation after LISA.
- The report provides implementation benchmarks for NICUs adopting LISA/MIST in similar settings, supporting protocols with universal caffeine and non-pharmacologic comfort without sedative premedication.

**Abstract:**

**Background**: Less invasive surfactant administration (LISA) can reduce exposure to mechanical ventilation in preterm infants, but factors associated with LISA failure in routine practice remain uncertain, particularly outside Europe. **Methods**: We performed a single-center retrospective cohort at King Abdulaziz Medical City, Riyadh (June 2023–June 2025). Inborn preterm infants at 26–34 weeks of gestation who received LISA as first-line surfactant therapy were included. The primary outcome was LISA failure, defined as intubation within 72 h for apnea, escalating oxygen requirement, or respiratory acidosis. Secondary outcomes included intraventricular hemorrhage (IVH), NICU length of stay, and other major morbidities. Multivariable logistic regression (gestational age as the anchor variable with a limited number of additional covariates) was used to identify predictors of failure and of IVH. Kaplan–Meier methods (log-rank test) were used to compare time to NICU discharge. **Results**: Forty-one infants were included (median gestational age: 30 weeks; median birth weight: 1300 g). LISA failure occurred in 39% of the cohort. Compared with infants with successful LISA, those who failed were more premature (median GA: 28 vs. 29 weeks; *p* = 0.009), had lower birth weight (1100 g vs. 1270 g; *p* = 0.011), higher IVH rates (38% vs. 8%; *p* = 0.020), and longer NICU stay (60 vs. 40 days; *p* = 0.041). Lower gestational age was the only independent factors associated with LISA failure (adjusted OR 1.44; 95% CI: 1.07–1.95; *p* = 0.018). LISA failure was independently associated with IVH (adjusted OR 10.08; 95% CI: 1.29–78.50; *p* = 0.027). Kaplan–Meier analysis showed significantly prolonged NICU stay among infants with LISA failure (*p* = 0.011). **Conclusions**: LISA is feasible in a high-acuity Middle Eastern NICU. However, failure—closely linked to lower gestational age—is associated with IVH and prolonged hospitalization. Careful patient selection and procedural planning are essential to optimize outcomes.

## 1. Introduction

Respiratory Distress Syndrome (RDS) is a major cause of morbidity and mortality in preterm neonates due to developmental deficiency of pulmonary surfactant and structural lung immaturity [1,2]. Traditional management has long relied on endotracheal intubation followed by surfactant administration and mechanical ventilation. However, this approach is associated with increased risks of ventilator-induced lung injury, Bronchopulmonary Dysplasia (BPD), and other complications related to invasive respiratory support [3,4].

In recent years, Less Invasive Surfactant Administration (LISA), also known as Minimally Invasive Surfactant Therapy (MIST) has gained prominence as an alternative method of surfactant delivery that avoids mechanical ventilation by administering surfactant through a thin catheter to spontaneously breathing infants maintained on non-invasive ventilation such as continuous positive airway pressure (CPAP) [5,6]. Clinical trials and systematic reviews have consistently shown that LISA reduces the need for mechanical ventilation, shortens the duration of respiratory support, and lowers the incidence of BPD compared to conventional endotracheal surfactant delivery [7,8,9]. These findings are further supported by international recommendations, including the European Consensus Guidelines [10].

Despite the growing body of evidence supporting the efficacy of LISA, its utilization remains limited and inconsistently adopted across many Neonatal Intensive Care Units (NICUs) in the Middle East, including Saudi Arabia. Most of the available evidence comes from randomized trials and observational studies conducted in European centers or countries with longstanding experience in standardized LISA protocols. In contrast, real-world data from high-acuity NICUs in the Middle East are sparse. Furthermore, variability in clinical thresholds for LISA initiation, patient selection, and the absence of national protocols may lead to inconsistent practice and limit reproducibility. It remains unclear how effective LISA is when applied in centers introducing the technique into routine care. Identifying factors associated with LISA failure is crucial for optimizing respiratory management, guiding appropriate candidate selection, and minimizing unnecessary procedural attempts.

This study addresses an important evidence gap by providing updated, single-center data on the implementation and outcomes of LISA in a tertiary NICU in Riyadh, Saudi Arabia. The primary objectives were to describe the demographic and clinical characteristics of preterm infants who received LISA, determine the incidence of LISA failure—defined as the need for intubation within 72 h—and identify factors associated with failure. In addition, the study evaluates key respiratory and clinical outcomes associated with LISA success versus failure. These findings contribute valuable regional evidence, may inform local protocol development, and support the safe and effective integration of LISA into routine neonatal care in similar healthcare settings.

## 2. Methodology

### 2.1. Study Design and Setting

This retrospective cohort study was conducted at the Neonatal Intensive Care Unit (NICU) of King Abdulaziz Medical City (KAMC), a tertiary academic center located in Riyadh, Kingdom of Saudi Arabia. The NICU is a high-capacity, level III facility with 50 intensive care cots and provides advanced care for extremely preterm and critically ill neonates. The unit is continuously staffed by consultant neonatologists, neonatal fellows, and specialized respiratory therapists. The study covered a two-year period, from June 2023 to June 2025. This study reflects the program’s initial adoption period in our unit.

### 2.2. Eligibility Criteria

All preterm infants born between 26 and 34 weeks of gestation who received LISA as their first mode of surfactant administration were eligible for inclusion. Infants were excluded if they had major congenital anomalies, required endotracheal intubation prior to surfactant administration, or were outborn and transferred into the unit after the procedure. During the study window, infants <26 weeks’ gestation were typically intubated in the delivery room and therefore were not candidates for LISA in our unit.

### 2.3. Respiratory Support Strategy

We follow a standardized pathway for preterm RDS. NCPAP is started in the delivery room and continued postnatally. LISA is offered in the first hours of life to spontaneously breathing infants on NCPAP with rising oxygen need (FiO_2_ ≥ 0.30) and clinical/radiographic evidence of evolving RDS. Mechanical ventilation is reserved for failure of non-invasive support—apnea not responsive to caffeine, persistent respiratory acidosis (pH < 7.20 and/or PaCO_2_ > 65 mmHg), recurrent desaturations despite support, or hemodynamic instability. Invasive modes include assist/control with volume guarantee and HFOV when indicated; non-invasive options include NCPAP, heated humidified high-flow nasal cannula, and nasal intermittent mandatory ventilation. Escalation and weaning follow unit criteria based on clinical assessment and blood-gas parameters.

Following LISA, endotracheal intubation is undertaken if any of the following occur: recurrent apnea/bradycardia requiring positive-pressure ventilation; requirement for FiO_2_ ≥ 0.40 to maintain target saturations; respiratory acidosis (e.g., pH < 7.20 and/or PaCO_2_ > 65 mmHg) or persistent hypoxemia despite non-invasive support; escalating work of breathing; or hemodynamic instability.

### 2.4. LISA Procedure Protocol

LISA was performed at the bedside under sterile conditions using the Surfcath™ catheter (Vygon, Paris, France), a semi-rigid yet flexible 6 Fr catheter specifically designed for surfactant administration. The catheter features a 20 cm length with a soft, angled 2 cm black distal tip for accurate tracheal placement, clear external markings for insertion depth confirmation, and transparent tubing for visual monitoring of surfactant delivery. Under direct laryngoscopy, the Surfcath™ was introduced into the trachea while the infant remained on nasal continuous positive airway pressure (NCPAP). Beractant (Survanta^®^, Chicago, IL, USA) was slowly administered at a dose of 100 mg/kg over 1–3 min. The procedure was performed by trained consultant neonatologists or neonatal fellows. No pharmacologic premedication was used to preserve spontaneous breathing; non-pharmacologic comfort measures (oral sucrose, swaddling, gentle laryngoscopy, and brief pauses) were employed. Infants were continuously monitored during and after the procedure for signs of bradycardia, apnea, desaturation, or respiratory deterioration requiring escalation of support.

### 2.5. Data Collection 

Data were collected retrospectively from the hospital’s electronic medical records using the Best-Care system. Collected variables included maternal and perinatal characteristics (antenatal corticosteroid administration, presence of gestational diabetes mellitus, preeclampsia, and prolonged rupture of membranes), neonatal demographic data (gestational age, birth weight, gender), and pre- and post-procedure respiratory support details, including duration of non-invasive ventilation (NCPAP, HHHHFNC, nasal cannula), and duration of invasive ventilation if applicable. The primary outcome was LISA failure, defined as the need for intubation and mechanical ventilation within 72 h of surfactant administration. Secondary outcomes included BPD at 36 wks. PMA, (IVH, any grade), culture-proven sepsis, total duration of hospitalization, and death before discharge. Caffeine citrate was routinely administered to infants < 34 weeks (loading 20 mg/kg, maintenance 5–10 mg/kg/day) before LISA whenever feasible; when LISA was initiated urgently, caffeine was given immediately afterward within the same clinical encounter.

### 2.6. Ethical Approval 

The study was reviewed and approved by the Institutional Review Board of King Abdullah International Medical Research Centre (KAIMRC), Ministry of National Guard Health Affairs, Riyadh, Saudi Arabia with IRB number (00000160825). As the study involved retrospective review of anonymized patient data, the requirement for informed parental consent was waived.

### 2.7. Statistical Analysis

All statistical analyses were conducted using IBM SPSS Statistics, Version 26.0 (IBM Corp., Armonk, NY, USA). The distribution of continuous variables was assessed using the Shapiro–Wilk test. As most continuous variables were non-normally distributed, they were summarized using medians and interquartile ranges (IQRs), and comparisons between groups were performed using the Mann–Whitney U test. Categorical variables were described using frequencies and percentages and compared using the chi-square test or Fisher’s exact test, as appropriate based on sample size.

The primary outcome was LISA failure, defined as the need for intubation within 72 h after surfactant administration. Infants were categorized into two groups based on this outcome (LISA success vs. LISA failure). Univariate analyses were conducted to identify candidate variables for multivariable modeling. Variables with a *p*-value < 0.1 in univariate analysis, in addition to those with established clinical relevance, were considered for multivariate logistic regression. Given the modest sample, we used simple models with a small number of key variables and emphasized effect sizes with 95% confidence intervals.

Two multivariable logistic regression models were developed. The first model examined factors associated with LISA failure and included gestational age, maternal antenatal steroid, infant gender, and maternal diabetes. Birth weight was excluded due to its collinearity with GA and the preference for GA as a more stable and routinely recorded variable in neonatal datasets. Adjusted odds ratios (aORs) with 95% confidence intervals (CIs) were reported. The second model evaluated predictors of any-grade intraventricular hemorrhage (IVH), with GA, antenatal steroids, and LISA outcome (success vs. failure) included as covariates. That model aimed to determine whether LISA failure independently increased the risk of IVH after adjusting for relevant perinatal factors. Finally, a Kaplan–Meier survival analysis was performed to assess length of NICU stay, comparing infants who experienced LISA failure versus those who did not. The log-rank test was used to assess statistical differences between survival curves. A two-tailed *p*-value < 0.05 was considered statistically significant for all analyses. Gestational age was analyzed as a continuous variable with associations reported per 1-week difference (95% CIs). Because caffeine exposure was uniform among infants < 34 weeks, it was not included as a covariate.

## 3. Results

A total of 41 preterm infants who received LISA were included in the study. The median gestational age was 30.0 weeks [(IQR: 28.0, 32.5], and the median birth weight was 1300 g [IQR: 1100, 1800]. Male infants comprised 61% of the cohort. Antenatal corticosteroids were administered to 68% of mothers. Maternal PROM was noted in 7% of mothers, while maternal hypertension was observed in 7% and maternal diabetes in 22% (Table 1).

Among the 41 preterm infants included in the cohort, the median duration of mechanical ventilation was 5 days [IQR: 4, 11] in those who failed LISA, while non-invasive ventilation was required for a median of 21 days [IQR: 6, 34] in the overall cohort. HHHFNC therapy was utilized in 68% of infants, with a median duration of 10 days [IQR: 8, 14]. Low-flow nasal cannula oxygen therapy was administered to 78%, with a median duration of 9 days [IQR: 7, 12]. Postnatal corticosteroids for respiratory support were given to 37% of infants. The LISA failure rate, defined as the need for intubation within 72 h of surfactant administration, was 39%. With respect to major neonatal morbidities, IVH of any grade occurred in 20% of infants. Bronchopulmonary dysplasia was diagnosed in 10%, and culture-confirmed sepsis was observed in 10%. The median length of NICU stay was 47 days [IQR: 38, 70] (Table 2). Severe ROP requiring treatment and surgical NEC were not observed (both zero events).

Among the 41 infants included, 25 (61%) had successful LISA, while 16 (39%) required intubation within 72 h following surfactant administration, indicating LISA failure. Infants who did not maintain non-invasive respiratory support after LISA had a significantly lower median gestational age (28 weeks [IQR: 26.5, 29.5]) and birth weight (1100 g [IQR: 900, 1400]) compared to those with successful LISA outcomes (29 weeks [IQR: 28, 30], *p* = 0.009; 1270 g [IQR: 1100, 1600], *p* = 0.011, respectively). Duration of low-flow nasal cannula use was longer in the group that required intubation (9 days [IQR: 6.5, 12]) than among those with sustained LISA success (7 days [IQR: 3, 11]; *p* = 0.036). NICU length of stay was also prolonged in the group that failed LISA (60 days [IQR: 46, 79]) versus the success group (40 days [IQR: 23, 60]; *p* = 0.041). Intraventricular hemorrhage occurred more frequently among infants who experienced LISA failure (38% vs. 8%; *p* = 0.020). No statistically significant differences were observed in rates of BPD, sepsis, or postnatal steroid use between the two groups (Table 3).

Multivariate logistic regression analysis was performed to identify independent predictors of the need for intubation within 72 h following LISA (Table 4). Among the evaluated variables, gestational age was the only factor that reached statistical significance. Each one-week reduction in gestational age was associated with a 44% increase in the odds of LISA failure (adjusted OR: 1.44; 95% CI: 1.07–1.95; *p* = 0.018), indicating that lower gestational maturity was the primary contributor to unsuccessful LISA. Other variables, including antenatal steroid exposure (adjusted OR: 1.05; 95% CI: 0.21–5.27; *p* = 0.950), male gender (adjusted OR: 1.64; 95% CI: 0.37–7.27; *p* = 0.513), and maternal diabetes (adjusted OR: 1.83; 95% CI: 0.35–9.55; *p* = 0.473), were not independently associated with LISA failure (Table 4).

Multivariate logistic regression analysis was conducted to evaluate factors independently associated with the occurrence of any grade of intraventricular hemorrhage (IVH) in the study cohort (Table 5). LISA failure was significantly associated with increased odds of IVH, with infants who required intubation within 72 h after LISA having over tenfold higher odds of developing IVH compared to those with sustained non-invasive respiratory support (adjusted OR: 10.08; 95% CI: 1.29–78.50; *p* = 0.027). This finding suggests that early respiratory compromise may contribute to the risk of IVH. Gestational age did not show a statistically significant association with IVH (adjusted OR: 0.83; 95% CI: 0.57–1.20; *p* = 0.324), though the direction of effect indicated a trend toward lower risk among infants born at higher gestational ages. Antenatal steroid exposure was also not significantly associated with IVH in this model (adjusted OR: 0.21; 95% CI: 0.02–2.45; *p* = 0.215) (Table 5).

**Figure 1:**
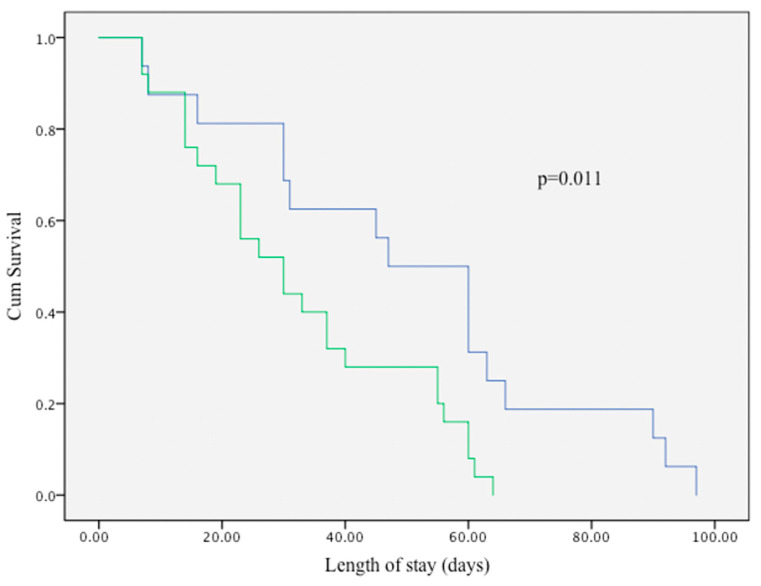
Kaplan–Meier Curve Comparing NICU Length of Stay by LISA Outcome.

Kaplan–Meier survival curve in Figure 1. compares the length of NICU stay between infants who experienced LISA failure and those who did not. Infants in the LISA failure group (blue line) demonstrated a significantly longer duration of hospitalization compared to those with successful LISA outcomes (green line). The difference between the two groups was statistically significant (log-rank test, *p* = 0.011), indicating an association between early LISA failure and prolonged NICU stay.

## 4. Discussion

This study was conducted to evaluate the clinical outcomes, risk factors, and factors associated with of failure among preterm infants who received less invasive surfactant administration (LISA) in a tertiary neonatal intensive care unit in Saudi Arabia. Although LISA is increasingly endorsed by international guidelines, its implementation and associated outcomes in the Middle Eastern context remain insufficiently documented. In this cohort, 39% of infants experienced LISA failure, defined as requiring intubation within 72 h of surfactant administration. The key finding was that lower gestational age was independently associated with a higher likelihood of LISA failure. Additionally, infants who failed LISA had significantly increased rates of IVH and prolonged NICU stays. These findings have important implications for clinical decision-making and healthcare resource planning in comparable neonatal settings. This study identified factors associated with intubation within a LISA-treated cohort and did not imply causality or compare LISA with alternative strategies. Lower gestational age showed a higher risk of intubation when modeled continuously, consistent with the known gradient by maturity.

Among the enrolled infants, the median gestational age was 30 weeks, and the median birth weight was 1300 g. The majority of infants were male, and antenatal steroid exposure was documented in more than two-thirds of the cohort. These baseline characteristics align with prior prospective studies on LISA cohorts from high-income settings and confirm that LISA is being offered to appropriately selected infants at moderate to late prematurity levels [5,6,10]. Our ANS uptake was lower than that reported in many LISA cohorts, which may partly account for differences in respiratory stability and early outcomes and should be considered when comparing effect sizes across studies. Caffeine citrate was routinely administered to infants < 34 weeks before LISA whenever feasible; when LISA was initiated urgently, caffeine was given immediately afterward within the same clinical encounter. Because caffeine enhances respiratory drive and supports spontaneous breathing, our protocol prioritizes administration before LISA; in urgent starts, immediate post-LISA dosing was used to avoid delaying surfactant delivery.

In terms of respiratory outcomes, the overall LISA failure rate of 39% is consistent with previously reported rates, which typically range from 25% to 40% depending on gestational age and selection criteria [8,9]. Importantly, infants who failed LISA were significantly more premature and had lower birth weights than those who succeeded. These differences were also reflected in their longer durations of low-flow nasal cannula use and prolonged hospitalization. These findings support the hypothesis that infants who fail LISA are more likely to experience hemodynamic and neurological instability, potentially due to the need for emergent intubation or limited physiological reserve. While our study focused on outcomes within a LISA-treated cohort, previous randomized trials—such as the one conducted by Kanmaz et al.—have demonstrated improved respiratory and neurological outcomes with LISA compared to the INSURE method. Their findings highlight the broader benefits of adopting LISA as the preferred approach for surfactant administration in spontaneously breathing preterm infants, particularly in reducing the need for mechanical ventilation and mitigating risks of severe intraventricular hemorrhage [11].

Emerging evidence also suggests that the risk factors for LISA failure may overlap with those for broader non-invasive ventilation (NIV) failure. A recent multicenter observational study reported that 15.6% of preterm infants initially supported with NIV required intubation within 72 h and experienced worse clinical outcomes, including IVH, pneumothorax, and increased mortality. Similar to our findings, lower gestational age was the most reliable factor associated with failure of NIV [12]. Together, these data highlight the critical role of the infant’s initial respiratory status—particularly in those with lower gestational maturity—in determining the likelihood of success with non-invasive surfactant strategies like LISA. Building on this concept, recent work has explored predictive tools that incorporate early physiologic markers to better identify infants at risk of LISA failure. For example, a Spanish cohort study demonstrated that factors such as intrauterine growth restriction, admission temperature, and lung ultrasound scores were strong factors associated with of CPAP failure following LISA, particularly in infants < 30 weeks’ gestation [13]. A predictive model incorporating these variables showed excellent discrimination (AUC ≈ 0.84), underscoring the potential value of real-time risk stratification. While our dataset did not include lung ultrasound or early physiologic indices, integrating such assessments into clinical practice could improve early decision-making and help tailor respiratory management in high-risk preterm infants.

Regression modeling further confirmed that gestational age was the only independent factors associated with LISA failure. For each one-week decrease in gestation, the odds of failure increased by approximately 44%. This observation echoes findings from Kribs et al., who demonstrated that LISA was most effective in infants born at or above 28 weeks’ gestation, and that extremely preterm infants are at greater risk of requiring escalation to invasive ventilation despite surfactant therapy [7]. Antenatal steroids, male gender, and maternal diabetes were not independently associated with LISA failure in our cohort, although point estimates and wide confidence intervals suggest that a larger sample may be required to further explore these associations.

In a separate regression model evaluating factors associated with IVH, LISA failure emerged as a significant risk factor. Infants who failed LISA had more than tenfold increased odds of developing IVH compared to those who did not. This finding highlights the potential link between early respiratory deterioration and cerebral injury, possibly mediated through fluctuations in cerebral blood flow and blood pressure during intubation or ventilatory transitions. Ballabh and colleagues have previously discussed how instability in cerebral perfusion during early life increases susceptibility to IVH in preterm infants, particularly when exposed to stressors such as mechanical ventilation [14]. Our findings support this pathophysiological pathway and highlight the need for careful monitoring of cerebral outcomes following LISA failure.

Analysis of NICU stay also revealed that infants who failed LISA had significantly longer hospitalizations. This was further demonstrated through a Kaplan–Meier survival analysis, which showed that time to discharge was significantly prolonged among those who failed LISA. These findings echo outcomes from Gupta et al., where Video Laryngoscopy-assisted LISA was associated with shorter NICU stays and reduced days on respiratory support compared to INSURE in infants ≥ 29 weeks’ gestation [15]. Furthermore, a recent review by Silveira et al. supports the broader association between LISA and reduced duration of ventilatory care, which often translates to earlier discharge [16].

While our findings provide important insights into the association between LISA failure and prolonged NICU stay, it is also instructive to compare these results with outcomes reported by more experienced centers and in larger cohorts. Centers with established LISA programs, such as those contributing to the German Neonatal Network, have reported favorable outcomes with first-line LISA implementation in extremely preterm infants (<27 weeks’ gestation), including reduced exposure to invasive mechanical ventilation, lower composite rates of death or BPD, and decreased incidence of severe IVH [17]. Adjunctive strategies such as early caffeine administration [18], video-laryngoscopy-assisted LISA [15,19], and the use of synchronized non-invasive ventilation [20] have also been associated with improved outcomes and reduced LISA failure rates. Collectively, these multicenter and interventional data highlight how institutional expertise, protocol standardization, and procedural refinements may contribute to optimized respiratory care and shorter hospitalization.

Our findings appear broadly aligned with this literature, particularly regarding the central role of gestational age and the association between LISA failure and adverse neonatal outcomes. These observations are further supported by a recent single-center study by Klein et al., which evaluated LISA use exclusively in extremely preterm infants born at <28 weeks’ gestation. Among 57 infants who received LISA in the delivery room, the reported success rate was 63%, with eligibility and success increasing with gestational age. LISA failure in that cohort was associated with increased risk of severe IVH, higher mortality, and reduced survival without major complications [21]. In our cohort, which included a broader gestational age range, the LISA success rate was 61%, closely aligning with Klein et al.’s findings. While their study focused on extremely preterm neonates, our results extend these observations to a wider population and strengthen similar concerns regarding the consequences of LISA failure.

While our study is based on a relatively modest sample size, it provides one of the first structured evaluations of LISA implementation in a high-acuity neonatal setting in the Middle East. Such data remain scarce despite growing global adoption of less invasive surfactant techniques. The findings offer clinically relevant information regarding the characteristics and outcomes associated with early LISA failure, particularly its strong association with gestational maturity and subsequent risk of intraventricular hemorrhage and prolonged hospitalization. These associations, although derived from a single center, are biologically plausible, consistent with previous international reports, and relevant to units with similar resources and population profiles. Furthermore, our study contributes to the evolving understanding of LISA performance across a broader gestational spectrum, extending observations from earlier studies that focused exclusively on extremely preterm infants. As LISA becomes increasingly integrated into neonatal practice worldwide, the need for contextualized, real-world data grows. Our experience demonstrates the feasibility of LISA without premedication and highlights the importance of procedural planning, patient selection, and local adaptation to optimize outcomes.

This study has several limitations that warrant consideration. The sample size was relatively small, which may limit the power to detect more subtle associations, particularly for secondary outcomes. The retrospective design also carries a risk of selection bias and unmeasured confounding, including clinician discretion in offering LISA. Lower ANS use in our program period is a contextual shortcoming that may attenuate LISA success relative to high-ANS settings; increasing ACS coverage is an active QI target in our unit. Additionally, our findings reflect the practices and thresholds of a single center and may not be generalizable to institutions with different staffing models, LISA techniques, or respiratory protocols. Nonetheless, these limitations are balanced by important strengths. The study provides detailed, systematically collected data on LISA outcomes in an underrepresented geographic region, contributing to the global literature with region-specific context. Our findings should be interpreted as exploratory associations and validated in larger, multicenter cohorts. Future work will focus on evaluating LISA pathways for the most premature infants as protocols evolve.

## 5. Conclusions

This study demonstrated the feasibility of implementing LISA in a high-acuity NICU setting in Saudi Arabia, with clinical outcomes largely consistent with those reported in international studies. Lower gestational age emerged as the strongest independent factors associated with LISA failure, which was in turn associated with increased risk of intraventricular hemorrhage and prolonged NICU stay. These findings emphasize the need for careful patient selection and procedural proficiency to optimize LISA outcomes. Future studies should focus on validating these results in larger multicenter populations and developing predictive tools to support clinical decision-making and reduce the likelihood of LISA failure.

## Figures and Tables

**Table 1:** Baseline characteristics of the cohort.

Variable	Median (IQR), *n* (%)
Gestational Age (Weeks)	30 [28, 32.5]
Birth Weight (g)	1300 [1100, 1800]
Male Gender	25 (61%)
Antenatal Steroid	28 (68%)
Maternal Prolonged Rupture of Membranes (PROM)	3 (7%)
Maternal Diabetes	9 (22%)
Maternal Hypertension	3 (7%)

**Table 2:** Respiratory support, neonatal morbidities, and clinical outcomes in preterm infants receiving LISA (*n* = 41).

Variable	Median [IQR], *n* (%)
Duration of Mechanical Ventilation (Days)	5 [4, 11]
Duration of Non-invasive Ventilation (Days)	21 [6, 34]
HHHFNC Use and Duration	28 (68%); 10 [8, 14]
Nasal Cannula Use and Duration	32 (78%); 9 [7, 12]
Postnatal Steroids for Respiratory Support	15 (37%)
LISA Failure	16 (39%)
Intraventricular Hemorrhage (Any Grade)	8 (20%)
Bronchopulmonary Dysplasia	4 (10%)
Culture-Confirmed Sepsis	4 (10%)
Length of NICU Stay (Days)	47 [38, 70]

**Table 3:** Comparison of clinical characteristics, respiratory outcomes, and morbidities between infants who succeeded and failed LISA.

Variable	LISA Success-*n* = 25 (61%)	LISA Failure-*n* = 16 (39%)	*p*-Value
Gestational Age (Weeks)	29 [28, 30]	28 [26.5, 29.5]	0.009
Birth Weight (g)	1270 [1100, 1600]	1100 [900, 1400]	0.011
Gender (Male)	14 (56%)	11 (69%)	0.414
Antenatal Steroid	16 (64%)	12 (75%)	0.460
PROM	2 (8%)	1 (6%)	0.834
Maternal Diabetes	5 (20%)	4 (25%)	0.706
Maternal Hypertension	1 (4%)	1 (6%)	0.884
Duration of CPAP (Days)	11 [5, 17]	21 [5.5, 33.5]	0.202
Need for HHHFNC	18 (72%)	10 (63%)	0.524
Duration of HHHFNC (Days)	10 [4, 15]	10 [7.5, 13.5]	0.356
Need for Low-Flow NC	20 (80%)	12 (75%)	0.706
Duration of Low-Flow NC (Days)	7 [3, 11]	9 [6.5, 12]	0.036
Length of NICU Stay (Days)	40 [23, 60]	60 [46, 79]	0.041
Postnatal Steroids	8 (32%)	7 (44%)	0.446
IVH (Any Grade)	2 (8%)	6 (38%)	0.020
BPD at 36 wks. PMA	2 (8%)	2 (13%)	0.636
Sepsis	2 (8%)	2 (13%)	0.636

**Table 4:** Multivariate logistic regression predicting LISA failure.

Predictor	*p*-Value	Adjusted OR (95% CI)
Gestational Age (Weeks)	0.018	1.44 (1.07–1.95)
Antenatal Steroids	0.950	1.05 (0.21–5.27)
Male Gender	0.513	1.64 (0.37–7.27)
Maternal Diabetes	0.473	1.83 (0.35–9.55)

**Table 5:** Multivariate logistic regression predicting any grade of intraventricular hemorrhage.

Predictor	*p*-Value	Adjusted OR (95% CI)
Gestational Age (Weeks)	0.324	0.83 (0.57–1.20)
LISA Failure	0.027	10.08 (1.29–78.50)
Antenatal Steroids	0.215	0.21 (0.02–2.45)

## Data Availability

The datasets used and/or analyzed during the current study are available from the corresponding author on reasonable request due to privacy and ethical restrictions.

## Abbreviations

LISA	Less Invasive Surfactant Administration
RDS	Respiratory Distress Syndrome
BPD	Bronchopulmonary Dysplasia
IVH	Intraventricular Hemorrhage
NICU	Neonatal Intensive Care Unit
GA	Gestational Age
NCPAP	Nasal Continuous Positive Airway Pressure
HHHFNC	Heated Humidified High Flow Nasal Cannula
NC	Nasal Cannula
OR	Odds Ratio
CI	Confidence Interval
aOR	Adjusted Odds Ratio
